# Genetic Variant HLA-DRB1*0403 and Therapeutic Response to Disease-Modifying Therapies in Multiple Sclerosis: A Case-Control Study

**DOI:** 10.3390/ijms241914594

**Published:** 2023-09-27

**Authors:** Esteban Alejandro Gomez-Gaitan, Yessica Eleanet Garcia-Ortega, Ana Miriam Saldaña-Cruz, Betsabe Contreras-Haro, Jorge Ivan Gamez-Nava, Emilio Edsaul Perez-Guerrero, Cesar Arturo Nava-Valdivia, Sergio Gallardo-Moya, Alejandra Martinez-Hernandez, Laura Gonzalez Lopez, Blanca Esthela Rios-Gonzalez, Jazmin Marquez-Pedroza, Miriam Mendez-del Villar, Yussef Esparza-Guerrero, Alejandra Villagomez-Vega, Miguel Angel Macias Islas

**Affiliations:** 1 Pharmacology Doctoral Program, Physiology Department, University Center for Health Sciences, University of Guadalajara, Guadalajara 44340, Jalisco, Mexico; esteban.gomez2861@alumnos.udg.mx (E.A.G.-G.); ivangamezacademicoudg@gmail.com (J.I.G.-N.); sergio.gallardo@alumnos.udg.mx (S.G.-M.); alejandra.martinez3431@alumnos.udg.mx (A.M.-H.); lauraacademicoudg@gmail.com (L.G.L.); yussef.esparza3286@alumnos.udg.mx (Y.E.-G.); 2 Neurology Department, Western National Medical Center, Mexican Social Security Institute, Guadalajara 44340, Jalisco, Mexico; yessimx12@gmail.com; 3 Institute of Experimental and Clinical Therapeutics, Physiology Department, University Center for Health Sciences, University of Guadalajara, Guadalajara 44340, Jalisco, Mexico; ana.saldanac@academicos.udg.mx; 4 Department of Biomedical Sciences, Tonala University Center, University of Guadalajara, Tonala 45425, Jalisco, Mexico; betsabe.contreras@academicos.udg.mx (B.C.-H.); miriam.mendez@academicos.udg.mx (M.M.-d.V.); alejandra.villagomez5427@academicos.udg.mx (A.V.-V.); 5 Biomedical Research Unit 02, Mexican Social Security Institute, Guadalajara 44340, Jalisco, Mexico; 6 Institute of Biomedical Sciences, Department of Genetics and Molecular Physiology, University Center for Health Sciences, University of Guadalajara, Guadalajara 44340, Jalisco, Mexico; edsaul.perezg@academicos.udg.mx; 7 Department of Microbiology and Pathology, University Center for Health Sciences, University of Guadalajara, Guadalajara 44340, Jalisco, Mexico; cesar.navavaldi@academicos.udg.mx; 8 Family Medicine Unit No. 92, Mexican Social Security Institute, Guadalajara 44990, Jalisco, Mexico; blanca2282@hotmail.com; 9 Neurosciences Division, Western Biomedical Research Center, Mexican Social Security Institute, Guadalajara 44340, Jalisco, Mexico; jaz180688@gmail.com; 10 Neurosciences Departament, University Center for Health Sciences, University of Guadalajara, Guadalajara 44340, Jalisco, Mexico

**Keywords:** multiple sclerosis, therapeutic response, HLA-DRB1*0403

## Abstract

Multiple sclerosis (MS) is a chronic and demyelinating disease with an autoimmune origin, which leads to neurodegeneration and progressive disability. Approximately 30 to 50% of patients do not respond optimally to disease-modifying therapies (DMTs), and therapeutic response may be influenced by genetic factors such as genetic variants. Therefore, our study aimed to investigate the association of the HLA-DRB1*0403 genetic variant and therapeutic response to DMTs in MS. We included 105 patients with MS diagnosis. No evidence of disease activity based on the absence of clinical relapse, disability progression or radiological activity (NEDA-3) was used to classify the therapeutic response. Patients were classified as follows: (a) controls: patients who achieved NEDA-3; (b) cases: patients who did not achieve NEDA-3. DNA was extracted from peripheral blood leukocytes. HLA-DRB1*0403 genetic variant was analyzed by quantitative polymerase chain reaction (qPCR) using TaqMan probes. NEDA-3 was achieved in 86.7% of MS patients treated with DMTs. Genotype frequencies were GG 50.5%, GA 34.3%, and AA 15.2%. No differences were observed in the genetic variant AA between patients who achieved NEDA-3 versus patients who did not achieve NEDA-3 (48.7% vs. 43.1%, *p* = 0.6). We concluded that in Mexican patients with MS, HLA-DRB1*0403 was not associated with the therapeutic response to DMTs.

## 1. Introduction

Multiple sclerosis (MS) is the most frequent demyelinating inflammatory disease of the central nervous system. MS is the second cause of disability in young adults [1,2]. The worldwide MS prevalence ranges from 5.5 to 29.5%; a higher prevalence has been observed in North America and Western Europe, and it is less frequent in countries near Ecuador [3]. The precise etiology of MS is not yet well understood, but genetic, geographical, and environmental factors have been described [4]. Major histocompatibility complex (MHC) is the key susceptibility locus in MS. MHC class II appears to have a stronger association with MS than class I. About 200 polymorphisms have been studied for the susceptibility of developing MS [5]. This association has been fine-mapped to the extended haplotype HLA-DRB1, which was the most correlative. In a meta-analysis, Zhang et al. found that the DRB1*03 phenotype increases the risk of MS [6]. Additionally, Romero et al. found that DRB1*04 was not associated with the disease state, and these alleles are related to a worse prognosis when considering the time taken to reach severe disability [7]. Moreover, Zuñiga et al. using a high resolution typing, found that DRB1*04 is one of the most frequent alleles in Mexican admixed individuals [8].

Over the past 50 years, the MHC locus has been shown to influence many critical biological traits and individual susceptibility to autoimmune diseases [9]. Studies have also evaluated HLA-DRB1 haplotypes in other pathologies, demonstrating that HLA-DRB1 haplotypes are associated with radiological severity, mortality, and response to TNF-α inhibitor drugs [10].

The current management strategies focus on treating acute attacks, ameliorating symptoms, and reducing biological activity through disease-modifying therapies (DMTs) [11]. These pharmacological treatments modify the course of MS through the suppression or modulation of immune function, preventing the transition of autoreactive T and B lymphocytes across the blood-CSF barrier and through autoreactive lymphocyte depletion [12,13]. The efficacy of different DMTs has been demonstrated in various studies. However, approximately 30 to 50% of patients do not respond optimally to DMTs, not showing any response in some cases [14,15]. Early prediction of the response to treatment continues to be one of the main difficulties in treating patients with MS [16]. Multiple factors related to an adequate therapeutic response are unknown [17]. Henceforth, it is desirable to identify those factors that may be associated with DMT response, which could help to optimize disease management. Consequently, it is of great importance to study genetic factors. A substitution of a single amino acid, glycine-to-valine in position 86 of the epitope-binding alpha-helix of the HLA-DRB1*04 allele sequence might produce a breakdown of T-cell tolerance to antigens, leading to B-cell activation and differentiation to plasma-cells with overproduction of antibodies against the INFβ [18]. However, there are no previous studies assessing the relationship between the genetic variant HLA-DRB1*0403 and the therapeutic response to DMTs in MS patients. Therefore, it is necessary to explore the response to treatment and its relationship with the genes to personalize medicine in MS. The present study investigates the potential association of the genetic variant HLA-DRB1*0403 and therapeutic response to DMTs in MS.

## 2. Results

Table 1 presents the characteristics of 105 patients with MS who were assessed. Patients with MS had a mean age of 38.9 ± 10.2 years, and it was more frequently observed in the female sex. The mean disease duration was 8.8 ± 5.8 years. No disease activity was achieved in 86.7% of MS patients. Regarding the genetic characteristics, wild homozygote was observed in 50.5% of MS patients.

In data that are not shown in tables, we found, in the reference group, polymorphism genotypes, and it was found that 70% presented the wild homozygous genotype (GG), 30% presented the heterozygous genotype (GA), and none of the patients presented the polymorphic homozygous genotype (AA). Furthermore, the genotype distributions were consistent with HWE (*p* > 0.05).

Table 2 shows the comparison between MS patients who did not achieve NEDA-3 versus MS patients who achieved NEDA-3. We found a higher frequency of glatiramer acetate use in MS patients who achieved NEDA-3. No differences were observed in age (37.7 ± 10.1 vs. 39.1 ± 10.3, *p* = 0.6), disease duration (8.8 ± 5.4 vs. 8.8 ± 5.9, *p* = 0.9), and across the different treatments.

Table 3 compares the genotypes and allele frequencies of genetic variant HLA-DRB1*0403 between MS patients who achieved NEDA-3 and those who did not achieve NEDA-3. There were no significant differences between the frequency of genotypes in the comparison between MS patients who achieved NEDA-3 and those who did not achieve NEDA-3. Additionally, in the genetic models, no differences were observed in carriers of wild homozygote, heterozygote, or mutated homozygous for achieving NEDA-3, indicating no association between genetic variant HLA-DRB1*0403 and the therapeutic response to disease-modifying therapies in MS patients.

## 3. Discussion

The present study analyzes the potential association of the genetic variant HLA-DRB1*0403 and the therapeutic response to DMTs in MS. HLA-DRB1*0403 was not associated with therapeutic response to DMTs in MS. Wild homozygote (GG) was the most frequently observed in the Mexican mestizo population with MS. Our results indicate that the frequency of the HLA-DRB1*0403 (A allele) was 32.4% in MS patients from Western Mexico, which is higher than that observed in people with MS in the center of Mexico (10.78%) [19].

To date, to the best of our knowledge, this is the first study to observe the lack of association between this genetic variant and therapeutic response in MS patients. There are no studies in Mexico that report the results of the presence of this or another genetic variant in the mestizo population in Mexico, and the response to treatment with respect to DMTs in MS. The risk of non-response to DMTs was similar across the different disease-modifying therapies.

We studied this HLA-DRB1*0403 genetic variant as it is the most prevalent in our population in western Mexico. As we know, this HLADRB1 can promote immunogenic presentation share valine (V) at position 86 of the DRB1 groove, which may modulate peptide anchoring, and, therefore, the way the neurogenic peptide presents to the TCR of those Th1 cells involved in myelin essential protein (MBP) destruction, as reported in other studies. A substitution of a single amino acid, glycine-to-valine in position 86 of the epitope-binding alpha-helix of the HLA-DRB1*04 allele sequence might produce a breakdown of T-cell tolerance to antigens, leading to B-cell activation and differentiation to plasma-cells with overproduction of antibodies against the INFβ [18].

Previous studies have evaluated the frequency of HLA-DRB1*0403, observing an association between the genetic variant and the risk of developing multiple sclerosis in different populations [18,19,20,21]. In a meta-analysis, Zhang et al. reported that the heterogeneity of DRB1*04 frequencies was too high to merge data [22]. Therefore, DRB1*04 cannot be a good predictor for MS in Caucasians. However, the heterogeneity was markedly reduced by analyzing four-digit genotypes of DRB1*04 separately. Consequently, further studies should focus on four-digit genotypes of DRB1*04.

On the other hand, similar to our study, the association of HLA-DRB1*0403 with a non-therapeutic response has been reported in MS patients. Buck et al. reported, in a post hoc analysis in 941 patients treated with interferon β-1b, an increased risk for carriers of HLA-DRB1*04:01 (OR = 3.3) and carriers of HLA-DRB1*07:01 (OR = 1.8) for developing neutralizing antibodies to INFβ [23]. Moreover, Hoffmann et al. found an association between HLA-DRB1*0401-positive and HLA-DRB1*0408-positive patients and the development of antibodies to INFβ [18]. INFβ exhibits immunogenicity like other protein-based disease-modifying agents; up to 50% of patients may develop antibodies to INFβ, of which a significant proportion neutralize the activity of INFβ [24,25]. The development of antibodies to INFβ has been considered a significant factor contributing to clinical treatment failure. Our results differ from the observation of other populations. Mazdeh et al. reported a beneficial response, analyzed by reduced disease relapses and the stabilization of EDSS scores in the two years of follow-up, with interferon β-1a in patients with a HLA-DRB1*04 and HLA-A*03-DRB1*04 haplotype [26]. Furthermore, Romero Pinel et al. reported that the HLA-DRB1*04 allele was associated with a worse prognosis when considering the time taken to reach severe disability [7].

Our study observed an adequate response to DMTs in 86.7% of the patients, which supports new studies in search of other genetic variants that may be associated with an adequate response to treatment. NEDA-3 was achieved in 33.6% of the patients with MS. NEDA-3 was achieved in 30.9% of patients receiving INFβ, which agrees with the literature published in the West [27]. In another study where NEDA was defined as 12-week confirmed disability progression, no protocol-defined relapses, no new/enlarging T2 lesions, and no T1 gadolinium-enhancing lesions, the results indicated that NEDA was increased in MS patients treated with ocrelizumab vs. MS patients treated with IFNβ-1a (66.4% vs. 24.3%, *p* < 0.001) [28].

There are some strengths to our study. The most important aspect of our research is that we used a stricter definition to evaluate the therapeutic response to DMTs and we assessed this response using the outcome NEDA-3—currently the most effective tool to accomplish the goals of treatment in MS—compared to other studies where EDSS was utilized; we observed that NEDA-3 was suitable to detect the effectiveness of clinical interventions and to monitor disease progression. Rostein et al., in a meta-analysis, found no differences between NEDA-4 and NEDA-3 in the long-term disability progression assessment [29]. Another strength of our study is that we analyzed the therapeutic response with different available clinical practice DMTs.

However, our study has some limitations that must be considered. Other polymorphic sites could be implicated in the development of the therapeutic response to DMTs in MS. Further studies should include genetic expressions associated with these genotypes as well as the influence of other factors, such as interactions between diverse genetic variations. Linkage disequilibrium within other genetic variants should be analyzed. It has been suggested that the presence of a specific haplotype in the homologous chromosome could have an additive effect on the genetic susceptibility to response in MS. The NEDA-3 not-achieved group (Case group) has a relatively small number of patients.

Future investigations that could replicate the findings in this work are necessary to verify the biological precept of the plausibility of gene–environment interactions with therapeutic response to DMTs in MS. This study might reflect only the genetic characteristics of patients with MS from the Western population of Mexico. Therefore, a multicenter study including patients from other regions of Mexico should be performed to reflect the characteristics of the Western population in Mexico, and to increase the number of the patients in the NEDA-3 not-achieved group. On the other hand, although these multicenter studies are required, they probably would not modify our main conclusion that this variant has no significant influence.

## 4. Materials and Methods

### 4.1. Study Design

Case–control study.

### 4.2. Study Population

This study included 105 patients with multiple sclerosis recruited from an outpatient clinic of the West Medical Center in Guadalajara, Mexico, who were enrolled from January 2019 through to February 2020. Inclusion criteria were the following: (1) aged ≥ 18 years; (2) diagnosis of MS according to the 2017 criteria of McDonald [30]; (3) Mexican mestizo defined according to the Mexican National Institute of Anthropology and History (INAH) as “individuals who were born in Mexico, of the 3rd generation including their own and who were descendants of the original autochthonous inhabitants of the region and individuals who were mainly Spaniards” [31]; (4) treatment with DMTs, and a brain magnetic resonance image (MRI). To ensure a correct diagnosis, a neurologist performed the diagnosis. We performed a 1.5-tesla MRI with conventional T1, gadolinium-enhanced T1, T2, and fluid-attenuated inversion recovery (FLAIR) sequences and confirmed the presence of typical round hyperintense lesions in T2 and FLAIR sequences and hypointensity in T1 with or without enhancement distributed in a different location; the time of evolution differed for each patient [32]. Oligoclonal bands were not considered mandatory to support or exclude an MS diagnosis [33]. Patients with kidney or liver disease diagnoses, pregnancy, and other uncontrolled autoimmune or psychiatric diseases were excluded. Additionally, ninety-two healthy subjects (reference group) without MS diagnosis, no history of inflammatory or autoimmune disorders, and only one person per family were recruited from the Western population in Mexico.

### 4.3. Clinical Setting

This study included patients with MS referred from a tertiary care center (UMAE, Hospital de Especialidades, Centro Médico Nacional de Occidente [CMNO]) in Guadalajara, Mexico.

### 4.4. Clinical Assessments

All patients were assessed for a clinical and neurological evaluation. Disease activity was assessed using the outcome No Evidence of Disease Activity (NEDA-3), which was achieved when MS patients presented any of the composites. The first composite of NEDA-3 was the absence of clinical relapse. Relapse was defined as a single acute or subacute episode of focal neurologic symptoms, informed by the patients over at least 24 h, with or without recovery, in the absence of fever or infection. The second composite was the absence of disability progression. Functional systems were used to establish the disability score according to Kurtzke’s Extended Disability Status Scale (EDSS). Progression was defined as an EDSS increase of 1.5 or more if the baseline was zero, as an increase of 1 if the baseline was less than 5.5, and as an increase of 0.5 if the baseline was 5.5 or more. EDSS considers visual, brainstem, pyramidal, cerebellar, sensory, bowel and bladder, cerebral functions, and ambulation. The EDSS minimum score obtained is equal to 0 points, and the maximum score equal to 10 points. MS patients were classified with relative severe dysfunction when the score was ≥4 points [34]. The third composite was the absence of radiological activity. Active lesions were evaluated using magnetic resonance image (MRI). Radiological activity was defined as the occurrence of contrast-enhancing lesions on T1-weighted or new/enlarging hyperintense lesions on T2-weighted brain or spinal cord [24].

NEDA-3 was achieved when MS patients were presented with no relapses, no disability progression, and no radiological activity (Control group). Therefore, treatment failure (Case group) was defined as, within the last year of treatment, presenting any of the following composites: clinical relapse or disability progression or radiological activity.

### 4.5. Genotyping

Genomic DNA from 105 subjects was extracted from peripheral blood leukocyte samples using the modified Miller technique [35]. Genomic DNA was quantified using a Nanodrop Genomic, and the DNA was diluted in Tris-EDTA buffer to 20 ng/μL and placed in 200 μL propylene cryotubes (Eppendorf™, Hamburg, Germany). The genotyping of HLA-DRB1*0403 polymorphism was performed by quantitative polymerase chain reaction (qPCR) for allelic discrimination using TaqMan probes [36]. TaqMan Assay IDs C_27513057_20 was performed according to the manufacturer’s instructions (Applied Biosystems, Waltham, MA, USA); the StepOne™, Real-Time polymerase chain reaction (qPCR) system was employed for this purpose (Applied Biosystems). All results were independently analyzed by two investigators blinded to patient information. In the case of ambiguous results, the sample was examined a second time. The resulting genotypes for the genetic variants were classified into one of the following three categories: wild homozygote (GG), mutated homozygous (AA), and heterozygote (GA). In the present study, we adopted the following genetic models: dominant (GG vs. GA + AA) and recessive (GA + AA vs. GG).

### 4.6. Statistical Analysis

Qualitative variables were expressed as frequencies (%), while quantitative variables as means and standard deviation (SD). We identified genotype frequencies by direct counting. Allele frequencies were determined by counting from the observed genotype frequencies. Comparisons between means were computed using the independent sample Student’s *t*-test. Comparisons between proportions were carried out using the chi-square test (or Fisher’s exact test if required). Odds ratios (OR) and their 95% confidence intervals (95% CI) were calculated.

## 5. Conclusions

In conclusion, the HLA-DRB1*0403 genetic variant does not confer a risk to achieving a therapeutic response with DMTs in Mexican mestizo patients with MS.

The search for other genetic variants explaining the therapeutic response in MS patients treated with DMTs is ongoing.

## Figures and Tables

**Table 1:** Selected characteristics in multiple sclerosis patients.

Variables	Multiple Sclerosis n = 105
Age (years), mean ± SD	38.9 ± 10.2
Female, n (%)	70 (66.7)
Disease characteristics	
Disease duration (years), mean ± SD	8.8 ± 5.8
EDSS score, mean ± SD	2.9 ± 1.9
EDSS ≤ 4, n (%)	75 (71.4)
EDSS > 4, n (%)	30 (28.6)
NEDA-3 achieved, n (%)	91 (86.7)
NEDA-3 not achieved, n (%)	14 (13.3)
Disease-modifying therapies	
Glatiramer acetate, use, n (%)	39 (37.1)
Interferon beta, n (%)	30 (28.6)
Rituximab, n (%)	9 (8.6)
Fingolimod, n (%)	14 (13.3)
Azathioprine, n (%)	5 (4.8)
Natalizumab, n (%)	3 (2.9)
Dimethyl fumarate, n (%)	5 (4.8)
Genetic Variant HLA-DRB1*0403	
Genotype	
G/G, n (%)	53 (50.5)
G/A, n (%)	36 (34.3)
A/A, n (%)	16 (15.2)
Allele	
G, 2n = 142 (%)	142 (67.6)
A, 2n = 68 (%)	68 (32.4)

Quantitative variables are expressed as mean ± standard deviation (SD); qualitative variables are expressed in frequency and percentage (%); EDSS: Extended Disability Scale; NEDA: no evidence of activity disease.

**Table 2:** Comparison of clinical variables between the case group (NEDA-3 not achieved) and control group (NEDA-3 achieved).

	NEDA-3 Not Achieved (Case Group) n = 14	NEDA-3 Achieved (Control Group) n = 91	*p*
Female, *n* (%)	12 (85.7)	59 (53.7)	0.1
Age (years), mean ± SD	37.7 ± 10.1	39.1 ± 10.3	0.6
EDSS score, mean ± SD	3.6 ± 2.3	2.8 ± 1.9	0.2
Disease duration (years), mean ± SD	8.8 ± 5.4	8.8 ± 5.9	0.9
Disease-modifying therapies			
Glatiramer acetate, n (%)	2 (14.3)	37 (40.6)	0.05
Dimethyl fumarate, n (%)	1 (7.1)	4 (4.4)	0.5
Fingolimod, n (%)	3 (21.4)	11 (12.1)	0.3
Interferon beta, n (%)	4 (28.6)	26 (28.6)	1.0
Natalizumab, n (%)	1 (7.1)	2 (2.2)	0.3
Rituximab, n (%)	2 (14.3)	7 (7.7)	0.4
Azathioprine, n (%)	1 (7.1)	4 (4.4)	0.7

Quantitative variables are expressed as mean and ± standard deviation SD; qualitative variables are expressed in frequency and percentage (%); EDSS: Extended Disability Scale; NEDA: no evidence of activity disease.

**Table 3:** HLA-DRB1*0403 genetic variant as a predictor of therapeutic response in patients with multiple sclerosis treated with disease-modifying therapies.

Multiple Sclerosis (n = 81)	NEDA-3 Not Achieved (Case Group) n = 14	NEDA-3 Achieved (Control Group) n = 91	OR	95%CI	*p*
Genotypes					
GG, n = 53 (%)	8 (57.1)	45 (49.5)	-	-	
GA, n = 36 (%)	5 (35.7)	31 (34.1)	-	-	0.6
AA, n = 16 (%)	1 (7.2)	15 (16.4)	-	-	
Genetic models					
Dominant Model (GG vs. GA + AA)	-	-	0.73	0.23–2.28	0.80
Recessive Model (GG + GA vs. AA)	-	-	2.56	0.31–2.12	0.36
Alleles, 2n = 210	2n = 28	2n = 182			
G allele, 2n = 142 (%)	21 (75)	121 (66.5)	Referent		
A allele, 2n = 68 (%)	7 (25)	61 (33.5)	1.51	0.61–3.75	0.36

No evidence of activity disease; GG: wild homozygote; GA: heterozygote; AA: mutated homozygous; OR: odds ratio risk; 95% CI: 95% confidence interval; *p* value.

## Data Availability

The data are not publicly available due to reasons of sensitivity. The data presented in this study are available on request from the corresponding authors.

## References

[1] Noseworthy J.H., Lucchinetti C., Rodriguez M., Weinshenker B.G. (2000). Multiple Sclerosis. N. Engl. J. Med..

[2] Ramagopalan S.V., Sadovnick A.D. (2011). Epidemiology of Multiple Sclerosis. Neurol. Clin..

[3] Ehtesham N., Rafie M.Z., Mosallaei M. (2021). The global prevalence of familial multiple sclerosis: An updated systematic review and meta-analysis. BMC Neurol..

[4] Gasperi C., Andlauer T.F., Keating A., Knier B., Klein A., Pernpeintner V., Lichtner P., Gold R., Zipp F., Bergh F.T. (2020). Genetic determinants of the humoral immune response in MS. Neurol.—Neuroimmunol. Neuroinflam..

[5] Patsopoulos N.A., Baranzini S.E., Santaniello A., Shoostari P., Cotsapas C., Wong G., International Multiple Sclerosis Genetics Consortium (2019). Multiple sclerosis genomic map implicates peripheral immune cells and microglia in susceptibility. Science.

[6] Zhang J., Shi S., Zhang Y., Luo J., Xiao Y., Meng L., Yang X. (2017). Alemtuzumab versus interferon beta 1a for relapsing-remitting multiple sclerosis. Cochrane Database Syst. Rev..

[7] Romero-Pinel L., Pujal J.M., Martínez-Yélamos S., Gubieras L., Matas E., Bau L., Torrabadella M., Azqueta C., Arbizu T. (2011). HLA-DRB1: Genetic susceptibility and disability progression in a Spanish multiple sclerosis population. Eur. J. Neurol..

[8] Zúñiga J., Yu N., Barquera R., Alosco S., Ohashi M., Lebedeva T., Acuña-Alonzo V., Yunis M., Granados-Montiel J., Cruz-Lagunas A. (2013). HLA Class I and Class II Conserved Extended Haplotypes and Their Fragments or Blocks in Mexicans: Implications for the Study of Genetic Diversity in Admixed Populations. PLoS ONE.

[9] Matzaraki V., Kumar V., Wijmenga C., Zhernakova A. (2017). The MHC locus and genetic susceptibility to autoimmune and infectious diseases. Genome Biol..

[10] Samadzadeh S., Tabibian E., Sabokbar T., Shakoori A., Dehgolan S.R., Armaki S.A., Aslanbeigi B., Abolfazli R. (2015). HLA-DRB1 does not have a role in clinical response to interferon-beta among Iranian multiple sclerosis patients. J. Neurol. Sci..

[11] McGinley M.P., Goldschmidt C.H., Rae-Grant A.D. (2021). Diagnosis and Treatment of Multiple Sclerosis. JAMA.

[12] Hauser S.L., Cree B.A.C. (2020). Treatment of Multiple Sclerosis: A Review. Am. J. Med..

[13] Bose G., Atkins H.L., Bowman M., Freedman M.S. (2019). Autologous hematopoietic stem cell transplantation improves fatigue in multiple sclerosis. Mult. Scler. J..

[14] Río J., Nos C., Tintoré M., Borrás C., Galán I., Comabella M., Montalban X. (2002). Assessment of different treatment failure criteria in a cohort of relapsing-remitting multiple sclerosis patients treated with interferon β: Implications for clinical trials. Ann. Neurol..

[15] Stürner K.H., Borgmeyer U., Schulze C., Pless O., Martin R. (2014). A Multiple Sclerosis–Associated Variant of CBLB Links Genetic Risk with Type I IFN Function. J. Immunol..

[16] Ernstsson O., Gyllensten H., Alexanderson K., Tinghög P., Friberg E., Norlund A. (2016). Cost of Illness of Multiple Sclerosis—A Systematic Review. PLoS ONE.

[17] Kasper L.H., Reder A.T. (2014). Immunomodulatory activity of interferon-beta. Ann. Clin. Transl. Neurol..

[18] Hoffmann S., Cepok S., Grummel V., Lehmann-Horn K., Hackermueller J., Stadler P.F., Hartung H.-P., Berthele A., Deisenhammer F., Wassmuth R. (2008). HLA-DRB1*0401 and HLA-DRB1*0408 Are Strongly Associated with the Development of Antibodies against Interferon-β Therapy in Multiple Sclerosis. Am. J. Hum. Genet..

[19] Alaez C., Corona T., Ruano L., Flores H., Loyola M., Gorodezky C. (2005). Mediterranean and Amerindian MHC class II alleles are associated with multiple sclerosis in Mexicans. Acta Neurol. Scand..

[20] de la Concha E., Arroyo R., Crusius J., Campillo J., Martin C., de Seijas E.V., Peña A., Claverıa L., Fernandez-Arquero M. (1997). Combined effect of HLA-DRB1*1501 and interleukin-1 receptor antagonist gene allele 2 in susceptibility to relapsing/remitting multiple sclerosis. J. Neuroimmunol..

[21] Rojas O.-L., Rojas-Villarraga A., Cruz-Tapias P., Sánchez J.L., Suárez-Escudero J.-C., Patarroyo M.-A., Anaya J.-M. (2010). HLA class II polymorphism in Latin American patients with multiple sclerosis. Autoimmun. Rev..

[22] Zhang J., Chen N., Chen C., Zhang W., Zhu F. (2023). Characterization of the novel HLA-DRB1 allele, HLA-DRB1*04:328 in a Chinese individual. HLA.

[23] Buck D., Andlauer T.F., Igl W., Wicklein E.-M., Mühlau M., Weber F., Köchert K., Pohl C., Arnason B., Comi G. (2019). Effect of HLA-DRB1 alleles and genetic variants on the development of neutralizing antibodies to interferon beta in the BEYOND and BENEFIT trials. Mult. Scler..

[24] Schellekens H. (2002). Immunogenicity of therapeutic proteins: Clinical implications and future prospects. Clin. Ther..

[25] Sorensen P.S., Ross C., Clemmesen K.M., Bendtzen K., Frederiksen J.L., Jensen K., Kristensen O., Petersen T., Rasmussen S., Ravnborg M. (2003). Clinical importance of neutralising antibodies against interferon beta in patients with relapsing-remitting multiple sclerosis. Lancet.

[26] Mazdeh M., Taheri M., Sayad A., Bahram S., Omrani M.D., Movafagh A., Inoko H., Akbari M.T., Noroozi R., Hajilooi M. (2016). HLA genes as modifiers of response to IFN-β-1a therapy in relapsing-remitting multiple sclerosis. Pharmacogenomics.

[27] Zafar A., AlShamrani F.J.G. (2021). No evidence of disease activity-3 (NEDA-3) status in patients with relapsing remitting multiple sclerosis: Evidence from Saudi cohort receiving mainly Interferon. Mult. Scler. Relat. Disord..

[28] Havrdová E., Arnold D.L., Bar-Or A., Comi G., Hartung H.-P., Kappos L., Lublin F., Selmaj K., Traboulsee A., Belachew S. (2018). No evidence of disease activity (NEDA) analysis by epochs in patients with relapsing multiple sclerosis treated with ocrelizumab vs interferon beta-1a. Mult. Scler. J. Exp. Transl. Clin..

[29] Rotstein D., Solomon J.M., Sormani M.P., Montalban X., Ye X.Y., Dababneh D., Muccilli A., Saab G., Shah P. (2022). Association of NEDA-4 With No Long-term Disability Progression in Multiple Sclerosis and Comparison With NEDA-3: A Systematic Review and Meta-analysis. Neurol. Neuroimmunol. Neuroinflam..

[30] Thompson A.J., Baranzini S.E., Geurts J., Hemmer B., Ciccarelli O. (2018). Multiple sclerosis. Lancet.

[31] Sánchez-Serrano C. (1996). Mestizaje E Historia De La Población En México.

[32] Bakshi R., Thompson A.J., Rocca M.A., Pelletier D., Dousset V., Barkhof F., Inglese M., Guttmann C.R., Horsfield M.A., Filippi M. (2008). MRI in multiple sclerosis: Current status and future prospects. Lancet Neurol..

[33] Arrambide G., Tintore M., Espejo C., Auger C., Castillo M., Río J., Castilló J., Vidal-Jordana A., Galán I., Nos C. (2018). The value of oligoclonal bands in the multiple sclerosis diagnostic criteria. Brain.

[34] Kurtzke J.F. (2015). On the origin of EDSS. Mult. Scler. Relat. Disord..

[35] Miller S.A., Dykes D.D., Polesky H.F. (1988). A simple salting out procedure for extracting DNA from human nucleated cells. Nucleic Acids Res..

[36] Livak K.J. (1999). Allelic discrimination using fluorogenic probes and the 5′ nuclease assay. Genet. Anal..

